# Symposium on free radicals

**DOI:** 10.1155/S0962935193000237

**Published:** 1993


Mediators of Inflammation, 2, 143–147 (1993)

### Human parathyroid hormone fragment stimulates the *de novo* synthesis of prostaglandin endoperoxide synthase in chick calvaria

Chin-Yuh Yang,[1,CA] Ching-Liang Meng,[1] and Patrick Y-K. Wong*

[1]Department of Dentistry, Tri-Service General Hospital, National Defense Medical Center, 18 Sy-Yuan Road, Taipei 100, Taiwan, Republic of China

* *Present Address:* Department of Physiology, New York Medical College, Valhalla, New York 10595, USA

[CA] Corresponding Author

**THE human parathyroid hormone N-terminal fragment [hPTH-(1–34)] increases the conversion of exogenous unsaturated fatty acids to prostaglandins (PGs) in calvarial homogenates. Enzyme activities were completely blocked by indomethacin ($5 \times 10^{-7}$ M), a PG synthase inhibitor, and actinomycin D (5 $\mu$M), an inhibitor of transcription, by binding to DNA. In addition, a potent inhibitor of protein synthesis, cycloheximide (10 $\mu$M), totally inhibited the stimulating effect of hPTH-(1–34) on prostaglandin endoperoxide synthase (PG synthase, EC 1.14.99.1). The stimulatory effect of hPTH-(1–34) on PG synthase was also reduced by the addition of stannous chloride. However, epidermal growth factor (EGF), platelet-derived activating factor (PDGF), and ionophore A23187 did not show the same stimulating effect as hPTH-(1–34) on PG synthase in calvaria. The results further demonstrated that PG synthase is a membrane-bound enzyme in chick calvaria. In this communication, evidence is presented that hPTH-(1–34) stimulates the *de novo* synthesis of PG synthase as demonstrated by the increased activity in calvarial homogenates and microsomes.**

**Key words:** Calvaria, PG synthase, PTH

### Introduction

Parathyroid hormone (PTH) is a single chain 84-amino acid peptide (molecular weight of 9 500) secreted by the parathyroid glands. However, the structural requirements necessary for full biological activity are virtually satisfied by the $NH_2$-terminal 34-amino acid fragment.[1] Bone and kidney are the two principal target organs affected by PTH. PTH stimulates the breakdown of phospholipids from the rat tibia which occurs at the same time as calcium mobilization[2] and stimulates phosphoinositide turnover in mouse osteoblasts.[3] In contrast, several reports have demonstrated that PTH stimulates the growth of bone[4,5] and cartilage.[6,7] Endogenous skeletal prostaglandin (PG) production may participate in bone resorption and bone formation.[8] PGs may not mediate the action of PTH on bone resorption.[9] However, PGs do show a very close relationship with the action of PTH on skeletal tissues.[10–13] It has been demonstrated that the human parathyroid hormone N-terminal fragment [hPTH-(1–34)] stimulates $PGE_2$ synthesis by chick calvaria in an organ culture. It appears that hPTH-(1–34) stimulates the bone cells to convert arachidonic acid to prostaglandin $E_2$ ($PGE_2$), but does not activate the release of stored arachidonic acid.[13]

Prostaglandin biosynthesis involves the initial conversion of arachidonic acid to cyclic endoperoxide intermediates designated $PGG_2$ and $PGH_2$ by prostaglandin endoperoxide synthase.[14] These relatively unstable compounds are then metabolized by other enzymes to form stable PGs, or are transformed into non-prostaglandin derivatives.[15] The mechanism by which hPTH-(1–34) increases the synthesis of $PGE_2$ in chick calvaria appears to be related to the activation of enzyme activity in the biosynthesis of prostaglandins.[13] It is important to investigate whether hPTH-(1–34) can stimulate the *de novo* synthesis of PG synthase in chick calvaria. In this report, it was found that hPTH-(1–34) increases the conversion of exogenous unsaturated fatty acids to PGs in calvarial homogenates and microsomes.

PG synthase is a membrane-bound enzyme which has been purified from sheep,[16,17] rat adipose tissue,[18] rabbit renal papilla,[19] bovine seminal vesicles,[20] and Swiss mouse 3T3 fibroblasts.[21] The enzyme possesses two enzymatic activities, a cyclooxygenase which catalyses the oxygenation of polyunsaturated substrates such as arachidonic acid to form $PGG_2$ and a peroxidase which can use a variety of electron donors to reduce $PGG_2$ to $PGH_2$. This report is the first study which demonstrated that PG synthase is a membrane-bound enzyme in skeletal tissue.

### Materials and Methods

*Materials:* Preincubated fertilized chick eggs were obtained from Miaoli Livestock Propagation Station, Taiwan Livestock Research Institute.

© 1993 Rapid Communications of Oxford Ltd

Synthetic human parathyroid hormone N-terminal 1–34 peptide [hPTH-(1–34), 3 000 IU/mg] was purchased from Bachem Inc. (Torrence, CA, USA). This material was dissolved in a solution containing 0.5% sodium chloride, 0.2% sodium acetate, and 1% bovine serum albumin, aliquoted and stored frozen at −70°C until use. Epidermal growth factor (EGF) and platelet derived growth factor (PDGF) were obtained from Collaborative Research Inc. (Two Oak Park, Bedford, MA, USA).

Labelled compounds, $^3$H-arachidonic acid (202 Ci/mmol), $^3$H-PGE$_2$ (184 Ci/mmol), $^3$H-PGF$_{2\alpha}$ (180 Ci/mmol), $^3$H-6-keto-PGF$_{1\alpha}$ (157 Ci/mmol), $^3$H-PGD$_2$ (192 Ci/mmol) and $^3$H-TXB$_2$ (180 Ci/mmol) were purchased from Amersham Corporation (Arlington Heights, IL, USA). Linear-K preadsorbent thin-layer chromatography plates were purchased from Whatman International Ltd. (Maidstone, England). A BioRad protein assay was obtained from BioRad Laboratories (Richmond, CA, USA). Octadecylsilyl (ODS)-silica columns (Sep-Pak C18 cartridges) were obtained from Waters Associates (Milford, MA, USA).

Trypsin and a penicillin–streptomycin solution were purchased from Gibco Lab. (Grand Island, NY, USA). Foetal calf serum (FCS) was purchased from Biofluids Inc. (Rockville, MD, USA). Fungizone was purchased from E. R. Squibb & Sons Inc. (Princeton, NJ, USA). Dithiothreitol, hydroquinone, methanol, ethanol, isooctane, and acetic acid were purchased from Merck (Darmstadt, Germany). Ethyl acetate was obtained from Mallinckrodt Inc. (Paris, KY, USA). BGJ$_b$ medium (Fitton–Jackson Modification), arachidonic acid, indomethacin, glutathione, cycloheximide, actinomycin D, stannous chloride, ionophore, haemin, sodium dihydrogen phosphate, sodium phosphate, Trizma hydrochloride, EDTA, sodium chloride, magnesium chloride, sodium bicarbonate, potassium phosphate, calcium chloride, and ascorbic acid were obtained from Sigma Corporation (St Louis, MO, USA).

*Methods: Preparation of calvarial homogenates:* Calvariae were dissected aseptically from 17-day-old chick embryos and cultivated as previously described by Yang *et al.*[22] Routinely, the paired bones were preincubated in an organ culture system for 24 h, then the medium was replaced with fresh medium containing different tested additions for appropriate time periods. The paired bones from 20 chicks were randomly assigned to the experimental groups.

After this preincubation, paired bones from each group were harvested, weighed, and resuspended in 4 ml of sodium phosphate buffer (10 mM NaH$_2$PO$_4$, 10 mM Na$_2$HPO$_4$, 100 $\mu$M dithiothreitol, pH 7.4) and then homogenized with a Polytron operated at full speed for 2 min. The homogenates were centrifuged at 400 × $g$ for 5 min at 4°C to remove large unminced fragments. The supernatant was removed and incubated with 10 $\mu$M cold arachidonic acid (AA) and 10 $\mu$Ci $^3$H-arachidonic acid in an equal volume of 0.2 M Tris-chloride buffer (pH 8.5) containing 2 mM glutathione, 1 mM hydroquinone, and 2 $\mu$M haemin at 37°C for 10 min. The reaction was terminated by the addition of 2 vol. of ethanol and was ready for extraction and purification of eicosanoids.

*Isolation of microsomes from calvaria:* The procedures for isolating microsomes from calvariae were a modification of those of Stern and Vance.[23] All procedures were performed at 4°C unless otherwise indicated. After a preincubation period of 36 h with or without hPTH-(1–34), the calvariae were harvested and resuspended in an appropriate volume of sodium phosphate buffer (10 mM NaH$_2$PO$_4$, 10 mM Na$_2$HPO$_4$, 100 $\mu$M dithiothreitol, pH 7.4) and then homogenized with a Polytron operated at full speed for 2 min. Homogenates were centrifuged at 12 000 × $g$ for 15 min. The resulting supernatants were harvested and centrifuged at 100 000 × $g$ for 1 h to obtain cytosol and microsomal pellets. The supernatant was removed and the pellet was homogenized in fresh sodium phosphate buffer equivalent to one-half the volume of the total reaction mixture. The microsomes were further incubated with 10 $\mu$M cold arachidonic acid and 2 $\mu$Ci (9.4 nM) $^3$H-arachidonic acid in an equal volume of 0.2 M Tris-chloride buffer (pH 8.5) containing 2 mM glutathione, 1 mM hydroquinone, and 2 $\mu$M haemin at 37°C for 10 min. The reaction was terminated by the addition of 2 vol. of ethanol and was ready for extraction and separation of eicosanoids.

*Extraction and purification of eicosanoids:* The incubation precipitates from calvarial homogenates and microsomes were centrifuged at 800 × $g$ for 10 min at 4°C, and the supernatant layer was evaporated to aqueous phase. Ethanol was added to the residues to achieve a final concentration of 15% ethanol. The biological sample was further acidified to pH 3.5 with 1 M citric acid and applied to an ODS-silica column (Sep-Pak C18 cartridge). The Sep-Pak cartridge was attached to a 20 ml polypropylene Luerlok syringe and washed with 20 ml of ethanol and water successively. The column was washed with 20 ml of water, 20 ml 15% aqueous ethanol, and 20 ml of petroleum ether sequentially. The eicosanoids were eluted with 10 ml of ethyl acetate.[24] The collected samples were dried by evaporation under a stream of nitrogen. The residues were then reconstituted in 1 ml of ethanol and filtered through a 0.45 $\mu$m filter (Millipore).

The samples were again dried by evaporation under a stream of nitrogen, reconstituted in 50 $\mu l$ of chloroform/methanol (2:1, v/v) and prepared for thin-layer chromatography.

*Thin-layer chromatography:* Thin-layer chromatography was performed by a method described previously.[13] After TLC, the radioactivity of the spots was determined by directly counting the scraped spots using liquid scintillation spectroscopy. The activity of prostaglandin endoperoxide synthase was determined by measuring the conversion of exogenous arachidonic acid to $PGE_2$.

### Results

Previous experiments have shown that a 36-h preincubation with hPTH-(1–34) can activate endogenous $PGE_2$ synthesis and mineral mobilization in intact chick calvaria.[13,25] In this report, it was further found that human PTH-(1–34) at a concentration of 0.6 $\mu$g/ml stimulates PG synthase activity (about a three-fold increase) in chick calvaria. EGF at a concentration of 20 ng/ml and PDGF at a concentration of 20 mU/ml had no stimulatory effect on PG synthase activity (Fig. 1(A) and Table 1). Ionophore A23187 at a concentration of 10 $\mu$M also had no effect on this enzyme activity (Fig. 1(B) and Table 1).

The stimulatory effect of hPTH-(1–34) on PG synthase in chick calvaria, as well as the basal activity of PG synthase, was blocked by a cyclooxygenase inhibitor, indomethacin ($5 \times 10^{-7}$ M), and a transcription blocker, actinomycin D (5 $\mu$M). Moreover, the stimulatory effect of hPTH-(1–34) was completely abolished by a translation inhibitor, cycloheximide (10 $\mu$M) (Fig. 1(B) and Table 1), as it brought the activity of PG synthase back to the basal level. It appears that hPTH-(1–34) stimulates the *de novo* synthesis of PG synthase in calvaria.

Stannous chloride was also included in the experiments to reduce untransformed endoperoxide to prostaglandin $F_{2\alpha}$ ($PGF_{2\alpha}$).[14] The stimulatory effect of hPTH-(1–34) on PG synthase was blocked (statistically significant) by the addition of stannous chloride (1.9 mg/ml, 10 $\mu$M) in ethanol; however, the total enzyme activity level was still higher than the basal levels (Table 1).

We then tried to identify the location of PG synthase in chick calvaria. Human PTH-(1–34) stimulates the *de novo* synthesis of PG synthase in the microsomal fraction of the calvariae but not in

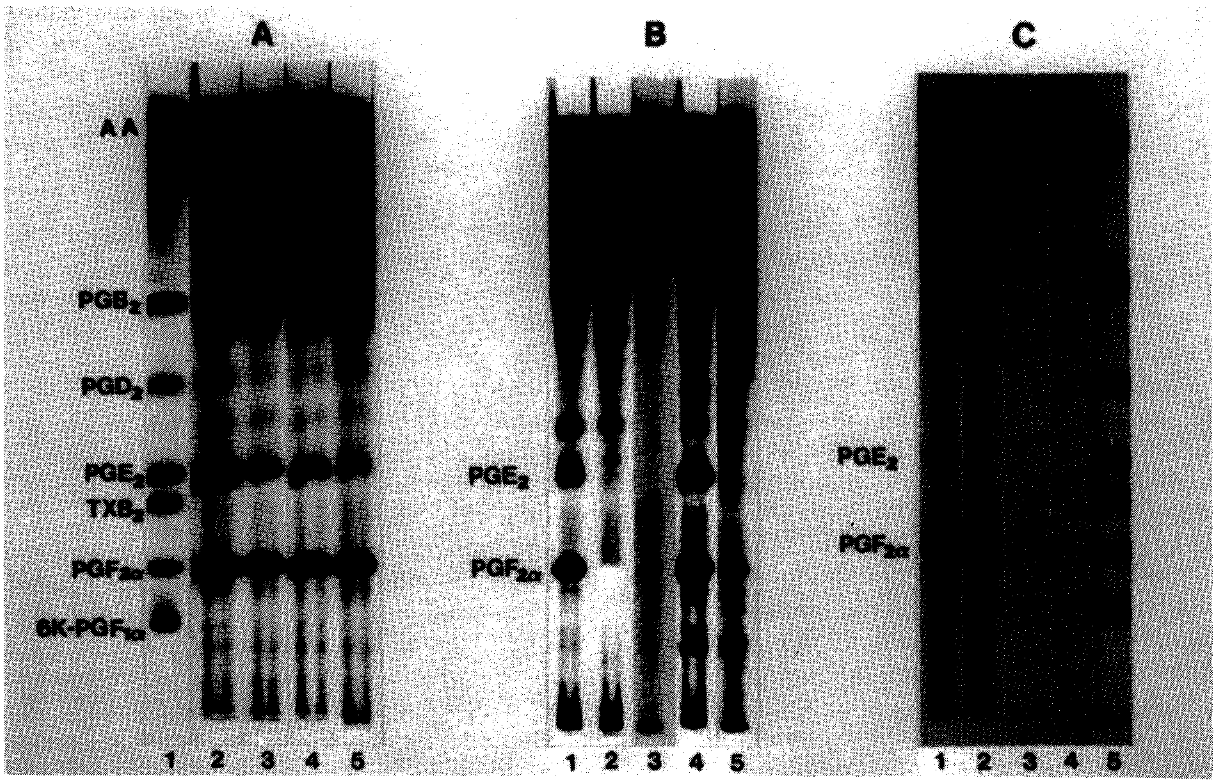

FIG. 1. (A) Autoradiograph of a TLC plate demonstrates the effect of hPTH-(1–34) and other additives on the conversion of arachidonic acid (AA) to prostanoids by chick calvarial homogenates. Lane 1: prostanoid standards. Lane 2: hPTH-(1–34) (0.6 $\mu$g/ml = $1.4 \times 10^{-7}$ M). Lane 3: EGF (20 $\mu$g/ml). Lane 4: PDGF (20 mU/ml). Lane 5: control. (B) Autoradiograph of a TLC plate demonstrates that the effect of blocking agents and effectors on PTH-induced synthesis of prostanoids by chick calvarial homogenates. Lane 1: hPTH-(1–34) (0.6 $\mu$g/ml) + cycloheximide (10 $\mu$M). Lane 2: hPTH-(1–34) (0.6 $\mu$g/ml) + actinomycin (5 $\mu$M). Lane 3: hPTH-(1–34) (0.6 $\mu$g/ml) + indomethacin ($5 \times 10^{-7}$ M). Lane 4: hPTH-(1–34) (0.6 $\mu$g/ml) + stannous chloride (1.9 mg/ml). Lane 5: ionophore A23187 (10 $\mu$M). (C) Autoradiograph of a TLC plate demonstrates that PG synthase activity stimulated by hPTH-(1–34) can only be recovered in the microsomal fraction of chick calvaria. Lane 1: hPTH-(1-34) (0.6 $\mu$g/ml), microsomal fraction. Lane 2: control, microsomal fraction. Lane 3: hPTH-(1–34) (0.6 $\mu$g/ml), cytosol fraction. Lane 4: control, cytosol fraction. Lane 5: prostanoid standards.

**Table 1.** The effect of hPTH-(1–34) and other additives on PG synthase activities in chick calvariae

| Additions | PG synthase activity, pg of $PGE_2$ synthesized per mg of protein per min |
|---|---|
| Control (No additions) | $50 \pm 4$ |
| hPTH (0.6 μg/ml) | $166 \pm 16$* |
| EGF (20 ng/ml) | $53 \pm 5$ |
| PDGF (20 mU/ml) | $57 \pm 7$ |
| hPTH + cycloheximide (10 μM) | $54 \pm 5$ |
| hPTH + actinomycin D (5 μM) | 0* |
| hPTH + INDO ($5 \times 10^{-7}$ M) | 0* |
| hPTH + $SnCl_2$ (1.9 mg/ml) | $117 \pm 12$*,** |
| Ionophore A23187 (10 μM) | $42 \pm 3$ |

The calvariae were incubated with different additions for 36 h, with the exception of the indomethacin (INDO) group which was incubated overnight. Indomethacin was preincubated for 1 h before adding hPTH-(1–34) in the hPTH + INDO group. After this preincubation, the bones were harvested and homogenized with a Polytron and centrifuged at 400 × *g* for 10 min at 4°C. The supernatant was harvested and incubated with 10 μM cold arachidonic acid and 10 μCi $^3$H-arachidonic acid for 10 min at 37°C. The prostanoids were then extracted with a Sep-Pak C18 cartridge and separated by TLC, and the enzyme activity was determined as described (for details of these procedures refer to Materials and Methods). Data are represented as the mean ± S.E. (disintegrations per min) for triplicate incubations of bone homogenates prepared from 20 chick calvariae. *Significantly different from control, as determined by Student's non-paired *t*-test ($p < 0.05$); ** Significantly different from hPTH treatment alone, as determined by Student's non-paired *t*-test ($p < 0.05$).

the cytosol fraction (Fig. 1(C)). These findings further suggest that PG synthase in chick calvaria in a membrane-bound protein.

### Discussion

The action of PTH on bone metabolism may involve several 'second messengers', including cAMP,[26] calcium,[27] the $Na^+$–$Ca^{2+}$ exchange mechanism[28] and prostaglandins.[29] In previous publications, it was demonstrated that hPTH-(1–34) stimulates calcium mobilization[25] and $PGE_2$ synthesis[13] in chick calvaria. In this communication, it has been further found that hPTH-(1–34) stimulates the *de novo* synthesis of PG synthase in chick calvaria. The authors speculate that PG synthase may be involved in the action of hPTH-(1–34) on bone resorption or bone formation which are coupling factors controlling bone metabolism.[5]

Since $PGE_2$ was the predominant eicosanoid produced by chick calvaria, the activity of PG synthase was determined by measuring the conversion of exogenous arachidonic acid to $PGE_2$. $PGF_{2\alpha}$ was the second major prostanoid to appear on TLC. Trace amounts of $PGB_2$, $PGD_2$, an unknown product that eluted before 6-keto-$PGF_{1\alpha}$ (more polar than 6-keto-$PGF_{1\alpha}$), an unknown product that eluted after $PGE_2$ (less polar than $PGE_2$), and an unknown product eluted after $PGD_2$ (less polar than $PGD_2$) were also detected.

Epidermal growth factor is a 53-amino acid polypeptide isolated from male mouse submaxillary glands, which stimulates $PGE_2$ synthesis in mouse calvaria.[30,31] Tashjian *et al.*[32] found that a low concentration of PDGF stimulates bone resorption via the enhanced local production of $PGE_2$. In previous observations, it was found that the use of different concentrations of EGF, PDGF, bradykinin and ionophore do not stimulate $PGE_2$ synthesis in chick calvaria. It was also noted that ionophore at a concentration of 10 μM has no deleterious effects on chick calvaria (unpublished data). Lack of responsiveness to these agonists may be due to a difference in species. In this study, the results further demonstrated that EGF, PDGF and ionophore have no stimulatory effects on the *de novo* synthesis of PG synthase in chick calvaria. On the other hand, it was found that hPTH-(1–34) does not have a stimulatory effect on PG synthesis in rat calvaria, whereas hPTH-(1–34) does show a minor effect (not statistically significant) in mouse calvaria (unpublished data). These observations all support the highly specific action of hPTH-(1–34) on chick calvaria.

The temporal sequence of PG synthase synthesized by human dermal fibroblasts can be separated into an early transcriptional stage and a subsequent translational stage.[33] The results in Table 1 indicate that the DNA transcription effect of hPTH-(1–34) on PG synthase can be blocked by the addition of actinomycin D, and the translational effect can be blocked by the addition of cycloheximide. At this moment, the authors are not able to conclude whether the stimulatory effect of hPTH-(1–34) on PG synthase occurs at the early transcriptional stage and/or the translational stage. It is possible that both stages are affected by hPTH-(1–34). However, it has been reported that hPTH-(1–34) stimulates the synthesis of DNA in the central bone of calvaria.[25] The biosynthesis of this enzyme in chick calvaria may be regulated by a specific gene. Isolation and determination of this specific gene might be a better way to clarify the real mechanism of hPTH-(1–34) acting on chick calvaria.

The incubation mixtures were treated with stannous chloride (1.9 mg/ml, 10 μM) in ethanol in order to reduce prostaglandin endoperoxide to $PGF_{2\alpha}$.[14] The results of these experiments do not show a complete reduction of $PGE_2$ production. The synthesis of both $PGE_2$ and $PGF_{2\alpha}$ may preclude the possibility of non-enzymic reduction of prostaglandin endoperoxide to these prostaglandins. The data also reveal that part of the stimulatory effect of hPTH-(1–34) on PG synthase is blocked by the addition of stannous chloride. The basal levels of this enzyme activity are not changed by $SnCl_2$, and a certain degree of the stimulatory effect of hPTH-(1–34) still remains (Table 1). In view of the partial inhibition by $SnCl_2$, it is quite possible that part of the increased activity may be due to induction of the PG endoperoxide E isomerase by hPTH-(1–34) that converts $PGH_2$ to $PGE_2$.

PG synthase contains both cyclooxygenase and peroxidase activities within a single protein. Cyclooxygenase component converts arachidonic acid to a hydroperoxy endoperoxide ($PGG_2$). The $PGG_2$ then reacts with PG synthase in a peroxidation reaction to give compound I (PGHS I), which subsequently converts to compound II (PGHS II). Native PG synthase is then regenerated.[34] Hsuanyu and Dunford[35] have demonstrated that the whole peroxidase cycle is rapidly completed (within seconds), and a mixture of compound I and compound II was achieved at a very early stage rather than pure compound I. Although the inducible form of PG synthase is now considered to be PGHS II, the authors are not able to conclude which form of PG synthase was activated by hPTH-(1–34) in the present study.

PG synthase has been discovered to be a membrane-bound protein in other tissues.[16–21] However, this enzyme has never been proved to be a membrane-bound protein in skeletal tissues. This communication strongly supports the view that PG synthase is a membrane-bound enzyme in chick calvaria.

### References

1. Tregear GW, van Rietschoten J, Greene E, *et al.* Bovine parathyroid hormone: minimum chain length of synthetic peptide required for biological activity. *Endocrinology* 1973; **93:** 1349–1353.
2. Cruess RL, Hong KC, Nakamura F. The effect of parathyroid hormone upon release of degradative enzymes from rat bone. *Proc Soc Exp Biol Med* 1979; **162:** 183–186.
3. Fardale RW, Sandy JR, Atkinson SJ, *et al.* Parathyroid hormone and prostaglandin $E_2$ stimulate both inositol phosphates and cyclic AMP accumulation in mouse osteoblast cultures. *Biochem J* 1988; **252:** 263–268.
4. Gaillard PJ, Herrman-Erlee MPM, Hekkelman JW, Burger EH, Nijweide PJ. Skeletal tissue in culture: hormone regulation of metabolism and development. *Clin Orthop Rel Res* 1979; **79:** 196–214.
5. Howard GA, Bottemiller BL, Turner RT, Rader JI, Baylink DJ. Parathyroid hormone stimulates bone formation and resorption into organ culture: evidence for a coupling mechanism. *Proc Natl Acad Sci USA* 1981; **78:** 3204–3208.
6. Suzuki F, Yoneda T, Shimonura Y. Calcitonin and parathyroid-hormone stimulation of acid mucopolysaccharide synthesis in cultured chondrocytes isolated from growth cartilage. *FEBS Lett* 1976; **70:** 155–158.
7. Burch WM, Lebovitz HE. Parathyroid hormone stimulates growth of embryonic chick pelvic cartilage *in vitro*. *Calcif Tissue Int* 1983; **35:** 526–532.
8. Raisz LG, Martin TJ. Prostaglandins in bone and mineral metabolism. In: Peck WA. ed. *Bone and Mineral Research*. Amsterdam: Elsevier Science Publishers BV, 1983; 286–310.
9. Gera I, Hock JM, Gunness-Hey M, *et al.* Indomethacin does not inhibit the anabolic effect of parathyroid hormone on the long bones of rats. *Calcif Tissue Int* 1987; **40:** 206–211.
10. MacDonald BR, Gallagher JA, Ahnfelt-Ronne I, *et al.* Effects of bovine parathyroid hormone and 1,25-dihydroxyvitamin $D_3$ on the production of prostaglandins by cells derived from human bone. *FEBS Lett* 1984; **169:** 49–52.
11. Raisz LG, Simmons HA. Effect of parathyroid hormone and cortisol on prostaglandin production by neonatal rat calvaria *in vitro*. *Endocrine Res* 1985; **11:** 59–74.
12. Feyen JHM, van der Wilt G, Moonen P, *et al.* Stimulation of arachidonic acid metabolism in primary cultures of osteoblast-like cells by hormones and drugs. *Prostaglandins* 1984; **28:** 769–781.
13. Yang CY, Gonnerman WA, Taylor L, *et al.* Synthetic human parathyroid hormone fragment stimulates prostaglandin $E_2$ synthesis by chick calvariae. *Endocrinology* 1987; **120:** 63–70.
14. Hamberg M, Svensson J, Wakabayashi T, Samuelsson B. Isolation and structure of two prostaglandin endoperoxides that cause platelet aggregation. *Proc Natl Acad Sci USA* 1974; **71:** 345–349.
15. Hamberg M, Samuelsson B. Prostaglandin endoperoxides. Novel transformations of arachidonic acid in human platelets. *Proc Natl Acad Sci USA* 1974; **71:** 3400–3404.
16. DeWitt DL, Rollins TE, Day JS, *et al.* Orientation of the active site and antigenic determinants of prostaglandin endoperoxide synthase in the endoplasmic reticulum. *J Biol Chem* 1981; **256:** 10375–10382.
17. Tanaka Y, Ward SL, Smith WL. Immunochemical and kinetic evidence for two different prostaglandin H–prostaglandin E isomerases in sheep vesicular gland microsomes. *J Biol Chem* 1987; **262:** 1374–1381.
18. Christ EJ, Nugteren DH. The biosynthesis and possible function of prostaglandins in adipose tissue. *Biochim Biophys Acta* 1970; **218:** 296–307.
19. Anggard E, Bohman SO, Griffin JE, *et al.* Subcellular localization of the prostaglandin system in the rabbit renal papilla. *Acta Physiol Scand* 1972; **84:** 231–246.
20. Miyamoto T, Ogino N, Yamamoto S, Hayaishi O. Purification of prostaglandin endoperoxide synthetase from bovine vesicular gland microsomes. *J Biol Chem* 1976; **251:** 2629–2636.
21. Rollins TE, Smith WL. Subcellular localization of prostaglandin-forming cyclooxygenase in Swiss Mouse 3T3 fibroblasts by electron microscopic immunocytochemistry. *J Biol Chem* 1980; **255:** 4872–4875.
22. Yang CY, Gonnerman WA, Menconi M, *et al.* Isolation and culture of two cell populations from chick calvaria by density gradient: Prostaglandin synthesis by the cells in culture and coculture. *Endocrinology* 1988; **123:** 2549–2556.
23. Stern PH, Vance DE. Phosphatidylcholine metabolism in neonatal mouse calvaria. *Biochem J* 1987; **244:** 409–415.
24. Nigam S. Extraction of eicosanoids from biological samples. In: Benedetto C, McDonald-Gilson RG, Nigam S, Slater TF, eds. *Prostaglandins and Related Substances: a Practical Approach*. Oxford: IRL Press Limited, 1987; 45–52.
25. Yang CY, Gonnerman WA, Polgar PR. The action of parathyroid hormone on bone metabolism may be regulated by endogenously synthesized prostaglandin $E_2$. In: Samuelsson B, Wong PYK, Sun FF, eds. *Advances in Prostaglandin, Thromboxane, and Leukotriene Research*. New York: Raven Press, 1989; **19:** 435–438.
26. Chase LR, Fedak SA, Aurbach GD. Activation of skeletal adenyl cyclase by parathyroid hormone *in vitro*. *Endocrinology* 1969; **84:** 761–768.
27. Marcus R, Orner FB. Parathyroid hormone as a calcium ionophore in bone cells: test of specificity. *Calcif Tissue Int* 1980; **32:** 207–211.
28. Krieger NS, Tashjian AH Jr. Parathyroid hormone stimulates bone resorption via a Na–Ca exchange mechanism. *Nature* 1980; **287:** 843–845.
29. Powles TJ, Easty DM, Easty GC., *et al.* Aspirin inhibition of *in vitro* osteolysis stimulated by parathyroid hormone and $PGE_1$. *Nature [New Biology]* 1973; **245:** 83–84.
30. Tashjian AH Jr, Levine L. Epidermal growth factor stimulates prostaglandin production and bone resorption in cultured mouse calvaria. *Biochem Biophys Res Commun* 1978; **85:** 966–975.
31. Byyny RL, Orth DL, Cohen S, Doyne ES. Epidermal growth factor: effects of androgens and androgenic agents. *Endocrinology* 1974; **95:** 776–782.
32. Tashjian AH Jr, Hohmann EL, Antoniades HN, Levine L. Platelet-derived growth factor stimulates bone resorption via a prostaglandin-mediated mechanism. *Endocrinology* 1982; **111:** 118–124.
33. Raz A, Wyche A, Needleman P. Temporal and pharmacological division of fibroblast cyclooxygenase expression into transcriptional and translational phases. *Proc Natl Acad Sci USA* 1989; **86:** 1657–1661.
34. Dietz R, Nastainczyk W, Ruf HH. Higher oxidation states of prostaglandin H synthase; rapid electronic spectroscopy detected two spectral intermediates during the peroxidase reaction with prostaglandin $G_2$. *Eur J Biochem* 1988; **171:** 321–328.
35. Hsuanyu Y, Dunford HB. Reduction of prostaglandin H synthase compound II by phenol and hydroquinone, and the effect of indomethacin. *Arch Biochem Biophys* 1992; **292:** 213–220.

ACKNOWLEDGEMENTS. The authors are greatly indebted to Ms W. H. Tien for her excellent technical assistance. This study was supported in part by grants from the National Science Council, Taipei, Taiwan, Republic of China (NSC78-0412-B016-49).

**Received 29 December 1992; accepted in revised form 3 February 1993**
